# The Impact of the Female Genital Microbiota on the Outcome of Assisted Reproduction Treatments

**DOI:** 10.3390/microorganisms11061443

**Published:** 2023-05-30

**Authors:** Giovanna Cocomazzi, Silvia De Stefani, Lino Del Pup, Simone Palini, Matteo Buccheri, Mariangela Primiterra, Natale Sciannamè, Raffaele Faioli, Annamaria Maglione, Giorgio Maria Baldini, Domenico Baldini, Valerio Pazienza

**Affiliations:** 1 Division of Gastroenterology, Fondazione IRCCS-Casa Sollievo della Sofferenza, 71013 San Giovanni Rotondo, Italy; g.cocomazzi@operapadrepio.it; 2 Clinica Nuova Ricerca, Via Settembrini 17/h, 47923 Rimini, Italy; silviadestefani@ymail.com (S.D.S.); primiterra.pma@nuovaricerca.com (M.P.); 3 Gynecological Endocrinology and Fertility, University Sanitary Agency Friuli Central (ASUFC), Via Pozzuolo, 330, 33100 Udine, Italy; info@delpupginecologia.it; 4 Ospedale “Cervesi” di Cattolica—AUSL Romagna Via Ludwig Van Beethoven, 1, 47841 Cattolica, Italy; simone.palini@auslromagna.it; 5 Instituto Bernabeu Via Castellana, 88, 30030 Martellago, Italy; matteobuccheri@gmail.com; 6 Gynecology and Obstetrics, IRCCS “Casa Sollievo della Sofferenza”, 71013 San Giovanni Rotondo, Italy; n.scianname@operapadrepio.it (N.S.); r.faioli@operapadrepio.it (R.F.); a.maglione@operapadrepio.it (A.M.); 7 IVF Center, Momò Fertilife, 76011 Bisceglie Via Cala dell’Arciprete, 76011 Bisceglie, Italy; gbaldini97@gmail.com (G.M.B.); dbaldini@libero.it (D.B.)

**Keywords:** vaginal microbiota, bacterial vaginosis, assisted reproduction technologies, endometriosis, infertility, pregnancy outcome, miscarriage

## Abstract

The vaginal microbiota plays a critical role in the health of the female genital tract, and its composition contributes to gynecological disorders and infertility. Lactobacilli are the dominant species in the female genital tract: their production of lactic acid, hydrogen peroxide, and bacteriocins prevents the invasion and growth of pathogenic microorganisms. Several factors such as hormonal changes, age of reproduction, sexual practices, menstrual cycle, pregnancy, and antimicrobial drugs use can cause imbalance and dysbiosis of the vaginal microbiota. This review aims to highlight the impact of the vaginal microbiota in Assisted Reproductive Technology techniques (ART) and it examines the factors that influence the vaginal microbiota, the consequences of dysbiosis, and potential interventions to restore a healthy female genital tract.

## 1. Introduction

**Figure 1:**
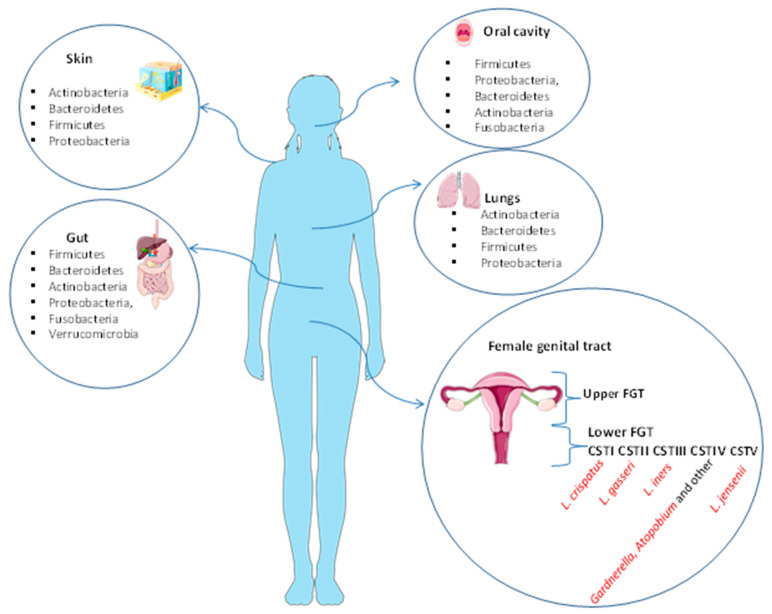
Human microbiota of the skin, gut, oral cavity, lungs, and female genital tract. The skin and the lung microbiota are composed of four different phyla: Actinobacteria, Bacteroidetes, Firmicutes, and Proteobacteria. The major bacteria present in oral microbiota are Firmicutes, Proteobacteria, Bacteroidetes, Actinobacteria, and Fusobacteria. The gut microbiota is composed of six phyla: Firmicutes and Bacteroidetes are the most abundant, followed by Actinobacteria, Proteobacteria, Fusobacteria, and Verrucomicrobia. The FGT is divided into the upper tract (endometrium and endocervix) and lower tract (vagina and ectocervix). The vagina is normally predominated by *Lactobacillus* spp. in the Community State Types (CST) I, II, III, and V. CST-IV is composed of a polymicrobial mix of obligate and facultative anaerobes bacteria (*Gardnerella*, *Atopobium* and other).

The microbiota plays an important role in human physiology and it consists of about one hundred trillion microbial cells that colonize our organism [1]. Ninety-five percent of the microorganisms constituting the entire human microbiota reside in the gastrointestinal tract, while the remaining five percent coexist in various organs and tissues such as the mouth, the lungs, the skin, and the vagina [2], where it performs specific functions. For instance, the skin and the lung microbiota play an essential role in protection against invading pathogens and are composed of four different phyla: Actinobacteria, Bacteroidetes, Firmicutes, and Proteobacteria [3,4]. The major bacteria present in oral microbiota are Firmicutes, Proteobacteria, Bacteroidetes, Actinobacteria, and Fusobacteria [5]. The gut microbiota is composed of six phyla: Firmicutes and Bacteroidetes are the most abundant, followed by Actinobacteria, Proteobacteria, Fusobacteria, and Verrucomicrobia [5]. The female genital tract microbiota (FGT) is generally divided into the upper tract (endometrium and endocervix) and lower tract (vagina and ectocervix) and therein plays a key role in preventing a number of urogenital diseases, such as bacterial vaginosis, yeast infections, sexually transmitted diseases, urinary tract infections [4,6,7], and HIV [8]. The FGT can influence potential fertility before and during assisted reproductive treatments (ARTs). The lower FGT is considered the “contact site” for the ingression of pathogens and it is estimated to have a bacterial load 10^2^–10⁴-fold higher than that seen in the upper FGT [6,9]. The tissues of the genital upper tract have been found to be sterile [10]. Lactobacilli are the dominant bacterial species in the lower tract (vagina) and they produce lactic acid to maintain an acid environment that prevents pathogen invasion and growth. Recent studies primarily use 16S rRNA gene sequencing for the identification of bacterial composition of the vagina (Figure 1).

It has been proved that there are at least five main types of vaginal microbiota that have been defined as “Community State Type” (CST) [11,12]. Four of these CSTs are dominated by members of the genus *Lactobacillus* spp., namely, *Lactobacillus crispatus* (CST-I), *L. gasseri* (CST-II), *L. iners* (CST-III), or *L. jensenii* (CST-V). CST-IV does not contain significant numbers of lactobacilli but it is rather composed of a polymicrobial mix of obligate and facultative anaerobes including bacteria belonging to the genera *Gardnerella*, *Atopobium*, *Mobiluncus*, *Prevotella*, and other taxa within the order of Clostridiales [13,14], which are often associated with bacterial vaginosis and obstetrical complications such as preterm birth [15]. Imbalance of vaginal microbiota can be caused by various factors such as hormonal changes, antibiotic use, and sexual behavior, leading to risk of vaginal dysbiosis, infertility, and preterm birth. It has also been ascertained that the microbiota is influenced by menstrual cycle, pregnancy, infections, methods of birth control, sexual behaviors [14], age, ethnic groups [11], and dietary intake [16,17]. Therefore, the composition of the vaginal microbiota could represent an important element for identifying the causes of female infertility classified as idiopathic, and for developing personalized therapeutic interventions such as probiotic use that may restore the balance of the vaginal environment and improve the success rates of assisted reproduction techniques.

## 2. The Community State Types (CSTs) of the Human Vaginal Microbiota

**Figure 2:**
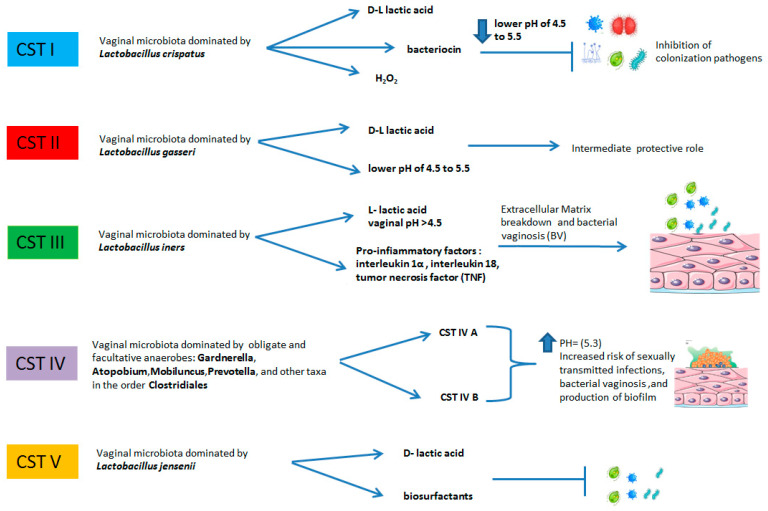
The relative abundances of bacteria in the following CSTs include: CST I: *L. crispatus* dominant, relatively stable. The vaginal pH = 4.5 due to the production of D-lactic acid. Production of hydrogen peroxide and bacteriocins inhibits invasion of microorganisms. CST II: *L. gasseri* dominant, similar to *L. crispatus* produces lactic acid and contributes to vaginal healthy milieu. CST has a more dynamic, intermediate protective role. CST III: *L. iners* dominant, pH > 4.5 elevated concentrations of pro-inflammatory markers, elevated risk of bacterial vaginosis. CST IV: no dominance, high diversity, and high proportions of anaerobic bacteria: *Prevotella*, *Dialister*, *Atopobium Vaginae*, *Gardnerella*, *Megasphaera*, *Peptoniphilus*, *Sneathia*, *Eggerthella*, *Aerococcus*, *Finegoldia*, and *Mobiluncus.* Elevated pH, damage to vaginal mucosa, probable production of biofilm and increased risk of sexually transmitted infections and bacterial vaginosis. CST V: *L. jensenii* dominant, more stable, protective role.

As stated above, female genital tract bacterial composition is classified into five main types of vaginal microbiota that have been defined as "Community State Type" (CST). The CSTs firstly proposed by Ravel et al. (2011) [11] and successively implemented by Gajer et al. (2012) [12] can be distinguished into two major categories: the first category is composed by CST I, CST II, CST III, and CST V and they are all dominated by *Lactobacillus* spp., while the second one includes CST IV and is further divided into the two sub-types CST IV-A and CST IV-B (Gajer et al., 2012) [12]. CST I is dominated by *Lactobacillus crispatus* and it is considered the most important for preventing infections such as bacterial vaginosis (BV) and for other health complications such us female infertility, preterm birth, and miscarriage [18]. CST II is dominated by *L. gasseri* which, similarly to *L. crispatus*, produces lactic acid and contributes to vaginal healthy milieu [19]. However, the role of *L. gasseri* is controversial because several studies show that its presence can negatively affect the fertility rate [20] and can predispose to bacterial overgrowth of the vagina in pregnancy [21]. Type III is dominated by *Lactobacillus iners*, which has been shown to be less protective against BV and pregnancy complications compared to other *Lactobacillus* species [22]. CST IV is characterized by low numbers of lactobacilli but is rather composed of a polymicrobial mix of obligate and facultative anaerobes including bacteria belonging to the genera *Gardnerella*, *Atopobium*, *Mobiluncus*, *Prevotella*, and other taxa within the order of Clostridiales. Type IV is associated with vaginal dysbiosis and it can make women more prone to recurrent infections. It is classified into two sub-types: CST IV-A and CST IV-B. CST IV-A contains moderate proportions of *L. crispatus*, *L. iners*, or other *Lactobacillus* spp., and various species of anaerobic bacteria such as *Anaerococcus*, *Corynebacterium*, *Finegoldia*, and some genera of *Streptococcus.* In contrast, CST IV-B is characterized by a higher proportion of the genus *Atopobium*, together with *Prevotella*, *Parvimonas*, *Sneathia*, *Gardnerella*, *Mobiluncus*, or *Peptoniphilus* [23]. CST V is deemed to be a healthy vaginal community state type, dominated by *Lactobacillus jensenii*, which creates a protective and stable environment vaginal similar to *L. crispatus* [24] (Figure 2).

Other microorganisms, especially Fungi (*Candida* and *Cryptococcus*), take part in the complex vaginal ecosystem together with other bacteria. These indigenous microorganisms exist in a mutualistic relationship with their human host. In this relationship, the host provides the nutrients needed to support bacterial growth, whereas the microorganisms play a protective role in preventing colonization of the host by potentially pathogenic organisms including bacterial vaginosis, yeast infections, sexually transmitted infections (STIs), and urinary tract infections [25]. The composition of the microbiota in women is influenced by various factors such as hormonal changes, age, menstrual cycle, sexual practices, antimicrobial drug usage, and pregnancy [26,27,28,29]. Romero et al. [30] have shown that the vaginal microbiota of pregnant women is different from that of non-pregnant women. In non-pregnant women, the vaginal microbiota is more stable, with minor fluctuations between CSTs and with higher abundance of *Lactobacillus vaginalis*, *L. crispatus*, *L. gasseri*, and *L. jensenii*, highlighting the importance of lactobacilli and their protective role during pregnancy [30]. The CSTs’ compositions could be used as an indicator of dysbiosis but also as predictor factor in ART outcome.

## 3. Role of Lactobacillus in the Female Genital Tract

Lactobacilli bacteria are group of bacteria that are characterized as Gram-positive, fermentative, facultative anaerobes, microaerophilic, acid-tolerant, and non-sporulating [31].

**Figure 3:**
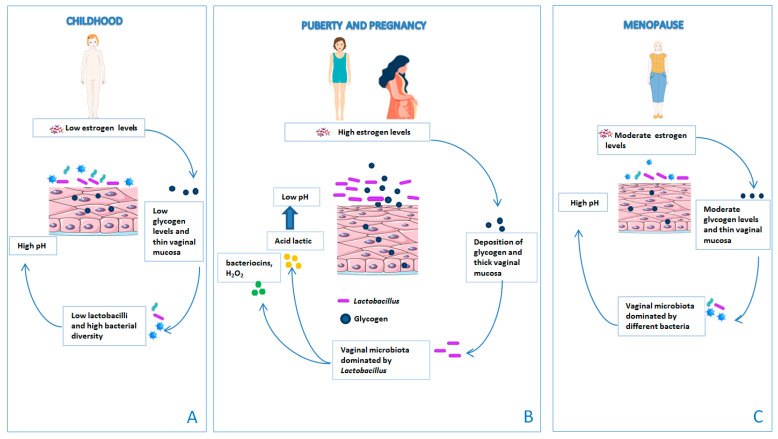
The vaginal microbiota changes throughout a woman’s life. (**A**) Prior to puberty, the production of estrogen and glycogen levels is low and the vaginal mucosa is thin. As a consequence, the vaginal pH increases and the vaginal microbiota is dominated by the presence of bacterial diversity and low *Lactobacillus* spp. (**B**) At the time of puberty, lactobacilli colonize the vagina due to the effect of high concentrations of estrogen on glycogen, which increases adherence of lactobacilli to vaginal epithelial cells. The vaginal mucosa is thick. (**C**) During menopause, there is a drastic reduction in the production of estrogen, with consequent dryness and atrophy of the vaginal epithelium. The reduction in the estrogen level decreases the glycogen content in the vaginal epithelium, causing a depletion of lactobacilli.

Most of these microorganisms have the ability to ferment lactose and other sugars producing lactic acid. *Lactobacillus* spp., regulates the balance of proinflammatory cytokines in vaginal secretions. It produces lactic acid, hydrogen peroxide, and bacteriocins that inhibit other vaginal microorganisms [25]. This balance can be rapidly altered during processes such as pregnancy, menstruation, sexual activity, and infections. Throughout a woman’s life, the vaginal microbiota undergoes major changes caused by factors associated with specific reproductive periods such as infancy, puberty, childbearing age, and menopause [32] (Figure 3). For instance, during early childhood, the vaginal pH is neutral or alkaline and the vaginal microbiota is colonized by *Corynebacterium* spp., *Staphylococcus epidermidis*, *E. coli*, and *Mycoplasma* spp. [16]. Prior to puberty, the production of estrogen and glycogen levels is low. As a consequence, the vaginal pH increases, the vaginal mucosa is thin, and the vaginal microbiota is dominated by the presence of bacterial diversity and low *Lactobacillus* spp. [33]. Lactobacilli colonize the vagina at the time of puberty due to the effect of high concentrations of estrogen on glycogen, which increases adherence of lactobacilli to vaginal epithelial cells. The vaginal mucosa is thick. In fact, during menopause there is a drastic reduction in the production of estrogen, with consequent dryness and atrophy of the vaginal epithelium. The reduction in the estrogen level decreases the glycogen content in the vaginal epithelium, causing a depletion of lactobacilli. Since glucose is not converted into lactic acid, there is an increase in the vaginal pH, which favors the growth of pathogenic bacteria, in particular enteric bacteria [34]. 

Disorders of the female genital tract such as bacterial vaginosis are characterized by the replacement of the *Lactobacillus* spp., in favor of a massive overgrowth of anaerobic and facultative organisms (*Gardnerella vaginalis*, *Atopobium vaginae*, *Bacteroides* spp., *Mobiluncus* spp., and genital mycoplasmas) and aerobic organisms, predominantly enteric commensals or pathogens [35]. A vaginal microbiota dominated by *Lactobacillus crispatus* (CST I) is often associated with a "healthy vagina", mainly due to its ability to produce lactic acid and bacteriocins, which make the vaginal environment inhospitable for some pathogenic microorganisms [16,25,30,31,32,33,34,35,36]. Bacteriocins are among the main antimicrobial molecules produced by lactic acid bacteria. They include a large family of peptides that act against bacteria even in small concentrations through different mechanisms such as the formation of pores in the bacterial membrane, and lead to cell death by potassium ions leakage or by membrane permeabilization and degradation of nucleic acids [37,38]. Furthermore, bacteriocins are susceptible to proteases, preventing the development of resistance to bacteria bacterial resistance. On the other hand, the lactobacilli form colonies that adhere to the cells of the vaginal epithelium, generating a physical barrier that precludes the adhesion of pathogens [39]. Furthermore, it has been observed that lactobacilli compete with *Candida albicans* and *Gardnerella vaginalis* for vaginal cell receptors and have a greater affinity with respect to pathogens, which allows them to counteract colonization by antagonistic species [40]. A vaginal environment dominated by *L. iners* (CST III) contributes to the rise of bacterial vaginosis (BV). The presence of *L. iners* is also associated with a vaginal environment prone to dysbiosis. Witkin et al. [41] suggest that this may depend on several factors, one of them being the ability of these *L. iners* to produce a distinct isomeric form of lactic acid (L-lactic acid) which is insufficient to inhibit the invasion of pathogenic bacteria. In fact, the absence of D-lactic acid seems to be involved in the breakdown of the extracellular matrix and consequently in the migration of pathogenic bacteria [41], while the presence of D-lactic acid or L-lactic acid influences matrix metalloproteinase-8 (MMP-8) concentrations. MMP-8, also known as neutrophil collagenase, is an enzyme involved in the turnover of the extracellular matrix and its degradation, and it induces a pro-inflammatory factors and ingression of bacteria [42]. Furthermore, the presence of *L. iners* is associated with elevated baseline values of pro-inflammatory factors such as macrophage migration inhibitory factor (MIF), interleukin-12p70, and tumor necrosis factor-alpha (TNF-α), which are responsible for activating inflammatory responses in the vagina [36]. Previous studies have already hypothesized that the "non-lactobacillus dominant" vaginal microbiota could be able to maintain a functional vaginal ecosystem thanks to the ability to preserve the production of lactic acid. However, recent studies have shown that 25% of women belonging to a different ethnic group do not have a lactobacilli-dominated microbiota and they are asymptomatic for vaginal disorder [11,16,17,18,19,20,21,22,23,24]. The variation in microbiota profiles may depend on genetic [43], geographic, social, and/or economic factors [44]. In particular, Hispanic and Black American women have a vaginal microbiota that lacks a significant number of lactobacilli species, but it is rich in facultative and strictly anaerobic microorganisms (*Atopobium*, *Corynebacterium*, *Anaerococcus*, *Peptoniphilus*, *Prevotella*, *Gardnerella*, *Sneathia*, *Eggerthella*, *Mobiluncus*, and *Finegoldia*), which may be able to maintain a functional vaginal ecosystem. Unlike lactobacilli that keep the vaginal pH around 4.5, these microorganisms are correlated with a higher vaginal pH (5.3–5.5). This suggests that the vaginal pH is not essential in maintaining the “health status of the vaginal microbiota” but that the latter may depend on other factors [45].

## 4. Vaginal Microbiota and Infertility

The World Health Organization (WHO) considers infertility as a pathology and defines it as the absence of conception after 12/24 months of regular unprotected sexual intercourse. This pathology can affect men, women, or both (couple infertility). Infertility in females can depend on several factors including genetic diseases, uterine pathologies, maternal age, and hormonal changes. Several studies suggest a correlation between infertility and the microbiota. For example, the presence of *E. coli* and other BV-associated pathogens in the female genital tract, such as *Atopobium vaginae*, *Sneathia sanguinegens*, *Sneathia amnionii*, *Chlamydia trachomatis*, *Mycoplasma genitalium*, and *Neisseria gonorrhoeae*, causes pelvic inflammatory disease (PID) [46]. Specifically, bacterial infections can affect fallopian tubes, ovaries, uterus, and pelvic peritoneum, and if not diagnosed and treated, they can become very dangerous up to the point of compromising female fertility. PID is the first cause of ectopic pregnancy due to the formation of fibrinous tissue inside the fallopian tubes, which prevents the fertilized egg from reaching the uterus during conception. The fertilized egg is then forced to be housed inside the tubes [47]. In addition, it has also been demonstrated that, in menstrual blood and in the fluid present in the peritoneal cavity, women with endometriosis have a higher concentration of Bacteria Gram− (*Escherichia coli*, *Gardnerella vaginalis*, *Proteus* spp., *Enterobacter* spp., *Neisseria gonorrhoeae*), Bacteria Gram+ (*Streptococcus* spp., *Staphylococcus* spp.), *Mycoplasma hominis*, and *Ureaplasma urealyticum* compared to controls [48,49]. Endometriosis is a chronic inflammatory disease that affects roughly 10% (190 million) of reproductive-age women, significantly affecting the quality of life of patients due to the symptoms it is often associated with [50]. It also influences female fertility by reducing the probability of reaching pregnancy both spontaneously and as a result of assisted reproduction treatments. In fact, endometriosis negatively affects follicular environment and consequentially oocyte maturation and competence, altering the levels of circulating gonadotropins and reducing the fertilization rate of oocytes [51,52]. The mechanisms according to which some microorganisms are involved in this pathology are still not understood. The most accredited hypothesis suggests that pathogens activate the immune response by binding with Toll-like receptors. Gram-negative microorganisms such as *Escherichia coli* possess lipopolysaccharide (LPS) on their cell wall, a bacterial endotoxin and marker of inflammation that promotes the onset and progression of endometriosis lesions through binding to the Toll-like receptor 4 [48].

Bacterial vaginosis (BV) is the most common genital tract disorder in reproductive-aged women. It is characterized by the absence of healthy bacteria, such as lactobacillus, and the presence of pathogenic bacteria (*Gardnerella vaginalis*, *Megasphaera* spp., *Atopobium vaginae*, *Dialister* spp.,) *Mobiluncus* spp., *Sneathia amnii*, *Sneathia sanguinegens*, *Porphyromonas* spp., and *Prevotella* spp. [13]. The diagnosis of BV can be conducted in a clinical setting using Amsel’s criteria or Nugent’s scoring system [53,54]. BV has been related to increased risk of infertility, particularly tubal infertility, and of second-trimester miscarriage in pregnant women undergoing in vitro fertilization (IVF) [55,56,57].

Tubal infertility occurs when the fallopian tubes are blocked due to diseases, damage, scarring, or obstructions that prevent the sperm from reaching the egg for fertilization, or that prevent an embryo from reaching the uterus for pregnancy. BV has been associated with preterm birth and miscarriage. The fallopian tubes connect each of the two ovaries to the uterus. The egg released from the ovary moves through these tubes toward the uterus and any present sperm also travels through the tubes, which is where fertilization of the egg normally occurs.

The correlation between BV infertility, miscarriage, and preterm deliveries is supported by the hypothesis of an excessive immune response. Kuon et al. [58], in a study on 243 patients with a history of three or more consecutive miscarriages, suggest the existence of a link between vaginal infections and the tendency to suffer repeated miscarriages. The analysis revealed a certain prevalence of patients positive to the presence of *Gardnerella vaginalis* (19%) and Gram-negative anaerobic bacteria (20.5%); furthermore, in 14.5% of the cases, the commensal bacteria lactobacilli were not present. In those patients, the number of NK cells was elevated, suggesting that potentially pathogenic microorganisms can stimulate an inflammatory response leading to systemic changes in immune parameters [58]. Several studies have demonstrated that the presence of BV, viral, and protozoan infections are linked to miscarriage [59]. In particular, the microorganisms involved in this process are *Streptococchi* [60], *Clamidia* [61], and *Listeria* [62]. The mechanisms according to which the infection caused by some microorganisms can lead to spontaneous abortion are not yet fully understood. Some microorganisms, such as Listeria, invade the maternal–fetal barrier by binding two proteins, internalin A and B, to the E-cadherin receptor at the basal and apical surface of the syncytiotrophoblast and cytotrophoblast villi of the placenta, causing spontaneous abortion following chorioamnionitis [62,63,64].

## 5. Role of Microbiota in Assisted Reproductive Technology (ART) Techniques

Assisted reproductive technologies (ART), as per the American Center for Disease Control (CDC) definition, are any fertility-related treatments in which eggs or embryos are manipulated. In vitro fertilization (IVF) techniques offer several opportunities to identify causes of infertility and difficulties in becoming pregnant. Several studies report that alterations of the vaginal microbiota correlate with a significant reduction in the pregnancy rate after IVF [65,66]. In a prospective study conducted by Koedooder R. et al. [67] on 303 women, it was demonstrated that, in addition to several factors such as age, number of oocytes, sperm quality, duration of infertility, and basal FSH levels, vaginal microbiota analysis can be used as a predictor of the failure to become pregnant after embryo transfer (ET). The study revealed that women with a high prevalence of *Lactobacillus crispatus* (CST I and CST III) have higher chances of becoming pregnant following ET in contrast to women with vaginal microbiota CST IV and CST V. In particular, the presence of *Gardnerella vaginalis* is associated with failure of pregnancy after ET [68]. Indeed, the contamination of the catheter used for embryo transfer has revealed that the presence of lactobacilli is associated with a better reproductive outcome [69], while the presence of Enterobacteriaceae and Staphylococci species is correlated with lower implantation rate, decreased pregnancy rates, and increased number of miscarriages [70].

Microorganisms have also been isolated in the upper genital tract, uterus [71], and ovarian follicle [72]. The uterine cavity has long been assumed to be sterile [73]. Several studies, according to some hypotheses, show that the bacteria reach the uterus through the cervical canal [71]. Moreno et al. [74] demonstrated the existence of an endometrial microbiota in women undergoing IVF. The 16s rRNA sequence analysis of paired samples of endometrial and vaginal fluid demonstrated that the bacteria present in the endometrium were not the same as those present in the vagina. An endometrial microbiota not dominated by *Lactobacillus*, with a high proportion of potential pathogens belonging to *Atopobium*, *Clostridium*, *Gardnerella*, *Megasphaera*, *Parvimonas*, *Prevotella*, *Sphingomonas*, or *Sneathia* genera, was associated with decreased live birth rates, implantation failure, and pregnancy loss. In the study [75] led by our group, two distinct profiles were observed associated with pregnancy outcome. *Lactobacillus iners*, Atopobium vagina, Peptonphylus lacrimalis, and timoniensis have been found in the cervix of women undergoing ART cycles associated with unfavorable pregnancy outcome. Conversely, Bifidobacterium, *Lactobacillus crispatus*, *Lactobacillus casei*, and *Lactobacillus delbrueckii* have been found in women with favorable pregnancy outcome.

The researchers aim to assess whether the use of antibiotics and probiotics actually improves IVF outcomes. We report, in the below paragraph, studies that evaluated the role of antibiotics, prebiotics, and probiotics in ART techniques, and their correlation with the success of pregnancy outcome.

## 6. Potential Antibiotic, Probiotic, and Prebiotic Uses to Restore Vaginal Health and Fertility

The use of antibiotics, prebiotics, and probiotics are considered useful approaches that could be used to maintain a healthy vaginal microbiota. The use of antibiotics as a prophylaxis to prevent bacterial contamination of the tip of the catheter used to transfer the embryos into the uterus is a practice already used in ARTs. The use of antibiotics may reduce female infertility, treating conditions such as bacterial vaginosis and pelvic inflammatory disease [76]. In a prospective study, it was shown that, in infertile women with chronic endometritis and with a history of repeated implantation failure (RIF), antibiotics improved the live birth rate after embryo transfer (ET) cycles [77]. However, antibiotics cannot be used for long-term treatments, due to the development of antibiotic-resistant [78] bacteria. Several studies have highlighted the efficiency of long- term probiotic administration in restoring vaginal microbiota as an alternative to the use of antibiotics. The FAO/WHO defines probiotics as "live microorganisms which when administered in adequate amounts confer a health benefit on the host". Probiotics can be helpful in restoring vaginal health and fertility by promoting a healthy balance of bacteria in the vagina. Mastromarino et al. [79] have demonstrated the effectiveness of using probiotics containing lactobacilli in the treatment of bacterial vaginosis [79]. In addition, a similar study showed a significant reduction in pro-inflammatory cytokines IL-1β and IL-6 [80]. A recent study observed the role of *Lactobacillus* as a prophylactic therapy in women with BV recurrence. Women were divided into two groups: group A received standard antibiotic treatment (metronidazole) while group B received antibiotics followed by *Lactobacillus rhamnosus*. Group B was more successful in preventing BV recurrence compared to group A that only received antibiotic therapy [81]. The use of probiotics for reproductive dysbiosis and infertility is continuously progressing and the therapeutic potential of probiotic therapy remains an opportunity in ART. However, Sirota et al. [82] reported that, when colonizing embryo catheter tips with *Lactobacillus crispatus* at the time of embryo transfer, the rates of implantation increase and the infection rates decrease [82]. Several scientific results have demonstrated benefits of probiotics on reproductive health outcomes: restoring the vaginal microbiota lactobacilli dominated bacterial vaginosis, rebalancing the gut–brain axis and resulting in an increase in sex hormones in women with PCOS [83], and reducing endometriotic lesions in a mouse model [84]. In fact, the interaction of bacteria between the gut and vagina stimulates immune responses resulting in an effect on the overall physiology of the host [85]. This interconnectedness between the gut and the vagina has been defined as the "gut–vagina axis". A key example is urinary infection by *Escherichia coli.* This can translocate from the gut to the urinary tract and later colonize, causing infections. Orally administered probiotics may improve beneficial effects to "gut–vagina axis" by preventing urinary infections and stimulating the systemic immune system [86]. In addition, the Norwegian mother-and-child cohort study highlighted that the use of milk containing probiotics during the first half of pregnancy reduced risk of spontaneous preterm delivery [87]. The colonization of probiotic bacteria is transient. They colonize the epithelium of the genital tract for a short period sufficient to restore the correct microbiota [88]. Some factors can negatively influence their colonization such as sexual intercourse and host immune system [89,90]. To improve their effectiveness, probiotics can be combined with prebiotics such as Lactoferrin [88]. Prebiotics are defined by the International Scientific Association for Probiotics and Prebiotics (ISAPP) as “a selectively fermented ingredient that results in specific changes in the composition and/or activity of the gastrointestinal microbiota, thus conferring benefit(s) upon host health” [91]. Feeding probiotics with prebiotics gives them the fuel they need to colonize the vaginal tract and improve their health. Scientific studies have reported the beneficial effects related to the restoration of microbiota colonization in reproductive sites, but there is still no clear evidence that probiotic and/or prebiotic therapy can modify the outcomes of assisted reproduction. Therefore, further studies are needed.

## 7. Conclusions

The female genital tract microbiota plays an important role in vaginal health and disease. Overall, *Lactobacillus* dominance is generally correlated with vaginal health, while a higher percentage of species such as *Gardnerella* and *Atopobium* is associated with dysbiosis, bacterial vaginosis, decreased live birth rates, implantation failure, and pregnancy loss. Host genetic factors, hormonal changes, age of reproduction, sexual practices, and pregnancy have been proven to alter vaginal microbial composition. BV, endometritis, PID, and inflammation play a role in contributing to infertility. Data suggest that FGT microbiota profiles could be exploited not only as markers of pregnancy outcome, but they also pave the way to new interventional strategies based on genital tract microbiota manipulation in order to increase the pregnancy rates in woman undergoing ART. Moreover, the evaluation of an FGT microbiota before ART and the possible combined use of probiotic/prebiotic and antibiotic therapies can improve IVF outcome. Further studies are urgently needed to obtain more information to develop effective therapies.

## Data Availability

Not applicable.
